# Prognostic significance of PET/CT for CAR T cell therapy in relapsed/refractory multiple myeloma

**DOI:** 10.1002/hem3.70159

**Published:** 2025-06-15

**Authors:** Patrick Born, David Fandrei, Song Yau Wang, Carmen Perez‐Fernandez, Luise Fischer, Jule Ussmann, Enrica Bach, Sandra Hoffmann, Klaus H. Metzeler, Marco Herling, Carmen Herling, Madlen Jentzsch, Andreas Boldt, Sabine Seiffert, Ronny Baber, Heike Weidner, Georg‐Nikolaus Franke, Timm Denecke, Osama Sabri, Uwe Platzbecker, Vladan Vucinic, Hans Jonas Meyer, Lars Kurch, Maximilian Merz

**Affiliations:** ^1^ Department of Hematology, Hemostaseology and Cellular Therapy University Hospital Leipzig Leipzig Germany; ^2^ Fraunhofer Institute for Cell Therapy and Immunology IZI Leipzig Germany; ^3^ Department of Radiology University Hospital Leipzig Leipzig Germany; ^4^ Department of Clinical Immunology University Hospital Leipzig Leipzig Germany; ^5^ Institute of Laboratory Medicine, Clinical Chemistry, and Molecular Diagnostics University Hospital Leipzig Leipzig Germany; ^6^ Leipzig Medical Biobank Leipzig Germany; ^7^ Department of Medicine III, Center for Healthy Aging Medical Faculty and University Hospital Carl Gustav Carus Dresden University of Technology Dresden Germany; ^8^ Department of Nuclear Medicine University Hospital Leipzig Leipzig Germany; ^9^ Myeloma and Cellular Therapy Services Memorial Sloan Kettering Cancer Center New York USA

## Abstract

PET/CT plays an important role in staging of multiple myeloma (MM) and detecting extramedullary disease (EMD); however, its role in patients treated with commercially available CAR T cell therapies is unclear. We evaluated 61 patients treated with CAR T cell products. In 53 patients, PET/CT was available before CAR T infusion, and 43 had follow‐up PET/CT on day 30. Findings from PET/CT were correlated to (CAR) T single‐cell dynamics, fitness and T cell receptor diversity after infusion, and serological markers of tumor burden and inflammation. Patients with bone‐independent EMD had inferior median progression‐free survival (PFS: 3 vs. 15 months, *p* = 0.01). Univariate and multivariate analysis showed that EMD but not the number of lesions or metabolic tumor volume (MTV) were associated with inferior PFS. High MTV was connected to higher baseline sBCMA and Interleukin‐6 levels, but not associated with hampered CAR T cell expansion or decreased fitness of the bystander T cell compartment. Follow‐up PET/CTs identified patients with metabolic complete remissions, which were associated with better PFS. PET/CT identifies patients with high risk of relapse after CAR T cell therapy.

## Graphical abstract

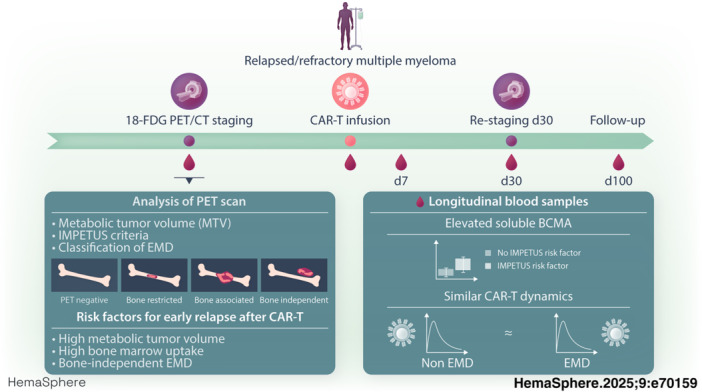

## INTRODUCTION

^18^F‐Fluorodeoxyglucose positron emission tomography–computed tomography (PET/CT) is pivotal in multiple myeloma (MM) for staging, monitoring treatment response, and detecting extramedullary disease (EMD).^1^, ^2^ Its prognostic significance has been described in patients undergoing autologous stem cell transplantation (ASCT).^3^, ^4^ However, its role in treatment with chimeric antigen receptor (CAR) T cell therapies for relapsed/refractory multiple myeloma (RRMM) has yet to be clearly defined. First reports indicate that the presence of EMD at the time of CAR T cell infusion is associated with adverse outcomes.^5^, ^6^, ^7^ Previously, two studies investigated the prognostic impact of PET/CT in CAR T cell therapy for MM. One performed longitudinal PET/CT assessments in patients treated with an academic CAR T cell product,^8^ and another correlated baseline PET/CT findings with soluble B‐cell maturation antigen (sBCMA) levels in patients predominantly treated with idecabtagene vicleucel (ide‐cel).^9^ However, structured longitudinal PET/CT evaluations using standardized criteria and their correlation with biological markers have not yet been fully explored. In this study, we analyzed the prognostic significance of longitudinal PET/CT imaging in 61 patients treated with ide‐cel or ciltacabtagene autoleucel (cilta‐cel), correlating imaging findings with serological remission assessments and longitudinal sBCMA measurements. Under the hypothesis that tumor load is associated with impaired (CAR) T cell function, we furthermore analyzed the impact on single CAR T cell expansion and T cell dynamics following infusion.

## METHODS

### Patients

We retrospectively studied patients with RRMM treated with commercially available CAR T products outside clinical trials. PET/CTs were performed before lymphodepletion and 4 weeks after CAR T treatment. Patients were followed‐up monthly to define remission according to the International Myeloma Working Group (IMWG) guidelines.^1^ Cytokine release syndrome (CRS) and immune effector cell‐associated neurotoxicity syndrome (ICANS) were graded following the American Society for Transplantation and Cellular Therapy (ASTCT) guidelines.^10^ The study received approval from the local ethics committee (361/22‐ek and LMB‐UCCL‐2022_06) and complied with the Declaration of Helsinki, with all participants providing written informed consent. To ensure objectivity, radiologists and nuclear medicine physicians were blinded to patient outcomes.

### Sample collection processing

Plasma blood samples were used to determine soluble B‐cell maturation antigen (sBCMA) and interleukin (IL)‐6 using commercially available ELISA, and absorbance was recorded using the FLUOstar OMEGA. The samples were collected at time points of leukapheresis, CAR‐T infusion, and days 7, 30, and 100 after intervention. Furthermore, we performed single‐cell RNA and TCR sequencing on peripheral blood mononuclear cells (PBMCs) collected on the day of leukapheresis, days 30 and 100 after CAR T cell re‐infusion as described previously.^9^, ^10^, ^11^ Single‐cell data were used to quantify PD1‐expression on T cell subsets and to calculate Shannon diversity indices of TCR sequences in CD4+ and CD8+ T cells of each patient and time point. CAR T cells were quantified by flow cytometry as described previously.^11^, ^12^, ^13^

### PET/CT imaging

Whole‐body [18F]fluorodeoxyglucose (FDG) PET/CT scans were evaluated by two nuclear medicine specialists and two radiologists. MM lesions were also quantitatively analyzed using Hybrid3D viewer software (HERMES Medical Solutions).^14^ To quantify total metabolic tumor volume (MTV) for each patient, focal lesions (FLs) were defined as regions of circumscribed standard uptake value (SUV) distinctly elevated above surrounding healthy tissue and baseline physiological glucose metabolism. MTV was calculated as the sum of the uptake volumes of all segmented pathological FLs identified in each patient. Due to the variable metabolic activity of MM lesions, a fixed SUV cutoff was not employed to determine MTV. Instead, cutoffs were manually adjusted to ensure accurate delineation of each lesion (see Supporting Information). Metrics including maximum SUV (SUVmax) and mean SUV (SUVmean) were recorded. Lesions were further classified into three groups: bone‐restricted, bone‐associated (involving cortical destruction and soft tissue infiltration), and bone‐independent extramedullary lesions, as described previously.^15^ Patients were stratified hierarchically based on lesion types and their associated prognostic implications, reflecting increasing adverse outcomes: (1) no lesions, (2) bone‐restricted lesions, (3) bone‐associated EMD with or without bone‐restricted lesions, and (4) bone‐independent EMD lesions with or without additional lesions.

In addition to our primary evaluation method, we also applied the Italian Myeloma criteria for PET USe (IMPeTUs) proposed by Nanni et al. to standardize PET/CT interpretation in MM.^16^ Scans were assessed using the 5‐point Deauville scale to evaluate diffuse bone marrow (BM) uptake as well as the focal bone and extramedullary lesion with the highest SUVmax. Furthermore, the CT component was analyzed to detect osteolytic lesions and associated fractures.

PET/CT was repeated at day 30 post‐infusion, a time point chosen based on existing evidence from lymphoma patients treated with anti‐CD19 CAR T cells demonstrating prognostic relevance,^17^ and coinciding with initial serological and BM assessments in patients with suspected complete remission.

### Statistical analyses

Statistical analyses were conducted using R version 4.3.1, and hypothesis tests were performed as indicated. Statistical significance was assumed at a *p* value cutoff of less than 0.05. For multigroup comparisons, all *p* values are given. For pairwise tests, only significant results are stated. Progression‐free survival (PFS) was defined as the duration from CAR T cell infusion to either disease progression or death from any cause and overall survival (OS) as time from CAR T cell re‐infusion to death from any cause. We assessed time‐to‐progression within various subgroups, presenting medians and 95% confidence intervals (CI) using the Kaplan–Meier estimator. Univariate and multivariate cox proportional hazard regression models (COX‐PH) were performed with the “coxph” package. Optimal cutpoint for continuous variables was determined by receiver‐operating characteristic (ROC) analysis and evaluated by area under the curve (AUC).

## RESULTS

### PET/CT findings in heavily pretreated patients before CAR T cell therapy

**Figure 1:**
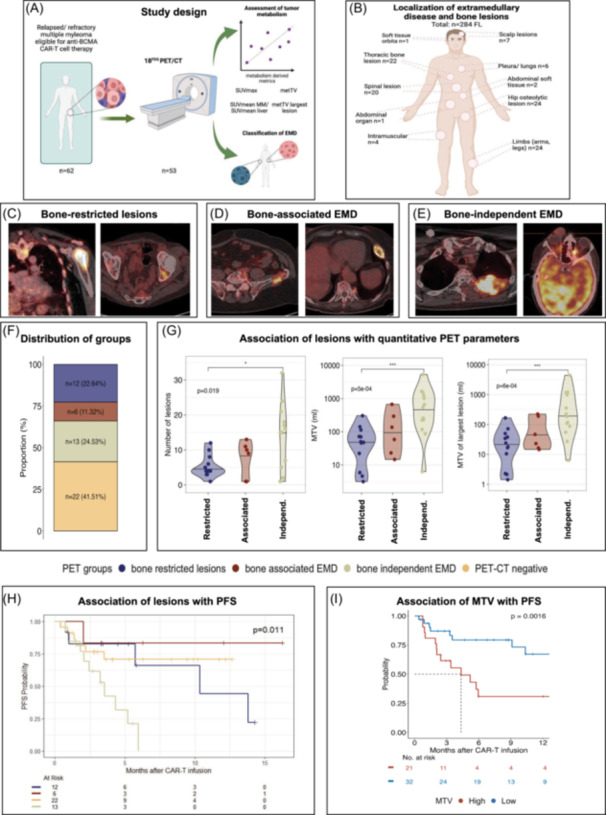
**PET/CT‐derived classification of extramedullary disease stratifies for outcomes after BCMA‐directed CAR T cell therapy in RRMM. (A)** Study workflow. **(B)** The number of patients with FL detected at different anatomical localizations in the cohort. Examples for the three different categories of FL are presented in **(C)**–**(E)**. **(C)** Two patients with intramedullary FDG‐avid focal lesion (FL) in the humerus (left) and acetabulum (right). **(D)** Examples for two patients with bone‐associated extramedullary disease (EMD) with cortical bone erosion in the iliac bone (left) and rib (right). **(E)** Examples of two patients with bone‐independent EMD of the pleural/thoracic soft tissue (left) and of the orbita (right). **(F)** Bar plot showing the numerical distribution of different types of FL in the entire cohort. Patients were hierarchically stratified by lesion type: (1) no lesions, (2) bone‐restricted lesions, (3) bone‐associated EMD with or without bone‐restricted lesions, and (4) bone‐independent EMD with or without additional lesions, reflecting worsening outcomes. **(G)** Violin plots showing correlation between types of FL (restricted to bone, bone‐associated EMD, and bone‐independent EMD) and FL counts (left), metabolic tumor volume (MTV, middle), and MTV of largest lesion (right). Multigroup comparison performed with Jonckheere‐Terpstra test. Progression‐free survival (PFS) according to the type of **(H)** FL and **(I)** MTV, with an optimal cuttoff of 54.3 mL. *p* values were obtained using the log‐rank test.

We retrospectively included 61 consecutive RRMM patients treated with BCMA‐directed CAR T cell therapy at our center from May 2021 until March 2024. Thirty‐four (55%) patients received idecabtagene vicleucel (ide‐cel) and 28 (45%) patients ciltacabtagene autoleucel (cilta‐cel). One patient received cilta‐cel after ide‐cel for 62 CAR T cell therapies. The cohort comprised heavily pretreated patients with a median of seven prior therapy lines [range 1–17], with 71% refractory to at least three drug classes, including PI, IMiD, and anti‐CD38 antibodies. Notably, 12 patients had penta‐refractory disease before CAR‐T therapy. Patient characteristics are summarized in Table 1. PET/CT was performed in 53 (85%) patients before lymphodepletion (Figure 1A). In line with previous results,^18^ 58% of patients with available PET/CT presented with FDG‐avid FL, with a median of 7 [range 1–32] FL in PET/CT positive cases. A total of 284 FLs were manually evaluated at baseline and during follow‐up, with their detailed anatomical distribution illustrated in Figure 1B. Among the cohort, 22 patients (42%) displayed no FL (PET/CT‐negative), while 12 patients (23%) showed only bone‐restricted FL (Figure 1C), 6 patients (11%) had additional bone‐associated EMD (Figure 1D), and 13 patients (25%) also exhibited bone‐independent EMD (Figure 1E,F). The number of FL, total MTV, and MTV of the largest lesion were significantly higher in patients with bone‐independent EMD compared to those with bone associated or bone‐restricted lesions (Jonckheere‐Terpstra test, *p* < 0.05, Figure 1G). Furthermore, there was a high correlation between the number of FL and MTV (*R* ^2^ = 0.37, *p* < 0.001, Figure S1).

**Table 1:** Patient characteristics.

	Total infusions (*n* = 62)
Product	
Ide‐cel	34 (55%)
Cilta‐cel	28 (45%)
PET/CT available	
Before	53 (85%)
After	43 (69%)
Age at CAR‐T infusion	
Median (IQR)	64 [58–68]
Range	31−75
Sex	
Male	35 (56%)
Female	27 (44%)
Prior lines of therapy	
Median (range)	7 (1–17)
EMD before CAR‐T (IMPeTUs)	
No	40 (75%)
Yes	13 (25%)
Pet classification	
Bone independent EMD	13 (25%)
Bone‐associated FL	6 (11%)
Bone‐restricted FL	12 (23%)
No FL	22 (42%)

### PET/CT identifies patients at high risk for relapse after CAR T cell infusion

There were no significant differences in depth of response after CAR T cell therapy based on PET/CT findings (Figure S2A). After a median follow‐up of 10 months [8–12], patients with bone‐independent EMD had significantly shorter median PFS (3 months [95% CI, 2–n.r.]) compared to those with bone‐associated EMD (15 months [15–n.r.]), bone‐restricted FL (13 months [5–n.r.]), or PET/CT‐negative patients (15 months [9–n.r.]; log‐rank test, *p* = 0.01, Figure 1H). Modes of progression varied by PET findings, with bone‐restricted lesions showing exclusively serologic progression, PET/CT‐negative cases exhibiting serologic (72%) and metabolic (28%) progressions, and metabolic progression predominating in bone‐associated (100%) and bone‐independent EMD (78%) cases (Figure S2B). An MTV cutpoint of 54.3 mL identified by ROC analysis (AUC = 0.7) identified patients with high MTV, who had significantly worse PFS (4 months vs. not reached, *p* = 0.001, Figure 1I). EMD status was not associated with other factors for adverse outcome after CAR T cell therapies, such as high‐risk cytogenetics, MyCARe and R‐ISS risk scores^19^ (Fisher's exact test, *p* = 0.8, Figure S3). Univariate Cox regression identified CAR T product (Cilta‐cel: HR = 0.28, *p* = 0.02), number of FL (HR = 1.06, *p* = 0.01), and bone‐independent EMD (HR = 3.9, *p* = 0.008) as PFS‐associated factors (Figure S4A). Multivariate analysis confirmed bone‐independent EMD as the sole independent predictor of worse PFS (HR = 5, *p* = 0.03 (Figure S4B).

### Impact of IMPeTUs classification on outcome after CAR T cell infusion

**Figure 2:**
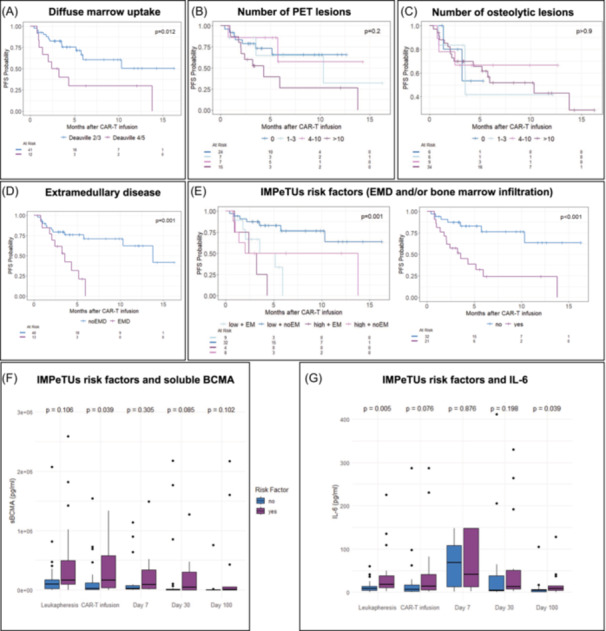
**IMPeTUs criteria and impact on progression‐free survival.** Progression‐free survival (PFS) analysis by **(A)** diffuse bone marrow uptake grouped by the metabolic activity level in comparison to the liver (Deauville 2 + 3 vs. 4 + 5), **(B)** number of PET‐positive focal lesions (FLs), **(C)** number of osteolytic lesions from CT, **(D)** presence of extramedullary disease (EMD), and **(E)** combination of metabolic risk factors diffuse BM uptake and EMD using the Kaplan–Meier estimator. *p* values were obtained using the log‐rank test. Longitudinal analysis of plasma level of **(F)** soluble BCMA and **(G)** IL‐6 compared in the previously defined risk groups. *p* values were calculated using the Wilcoxon‐rank sum test.

Next, we evaluated the prognostic relevance of pre‐infusion PET/CT findings according to the IMPeTUs criteria and their association with clinical outcomes following CAR T cell therapy. Diffuse BM uptake (>Deauville 3) was associated with an inferior outcome (log‐rank test, *p* = 0,012, Figure 2A). The number of PET‐positive FLs was significantly associated with PFS only in patients with >10 FLs, who had worse outcomes compared to PET‐negative patients (pairwise log‐rank, *p* = 0.025, Figure 2B). In our cohort, 62% of patients had >10 osteolytic lesions, but neither their number nor the presence of fractures affected PFS (Figure 2C). The presence of EMD according to IMPeTUs criteria was also associated with adverse outcome (Figure 2D) and combining risk factors from IMPeTUs (EMD and diffuse BM uptake) enabled us to identify patients with long‐term survival as well as early relapse after CAR T cell therapy (Figure 2E). Given the link between increased tumor burden at CAR T cell therapy and both—shorter survival and side effects—we analyzed the association between PET findings and CRS incidence. We found significantly higher baseline sBCMA (tumor burden, Figure 2F) and Il‐6 (mediator of CRS, Figure 2G) serum values in patients with EMD and/or diffuse BM infiltration in PET/CT. However, no significant differences in CRS occurrence or severity were observed based on PET/CT findings (Figure S5).

### Prognostic significance of PET/CTs performed after CAR T cell infusion

**Figure 3:**
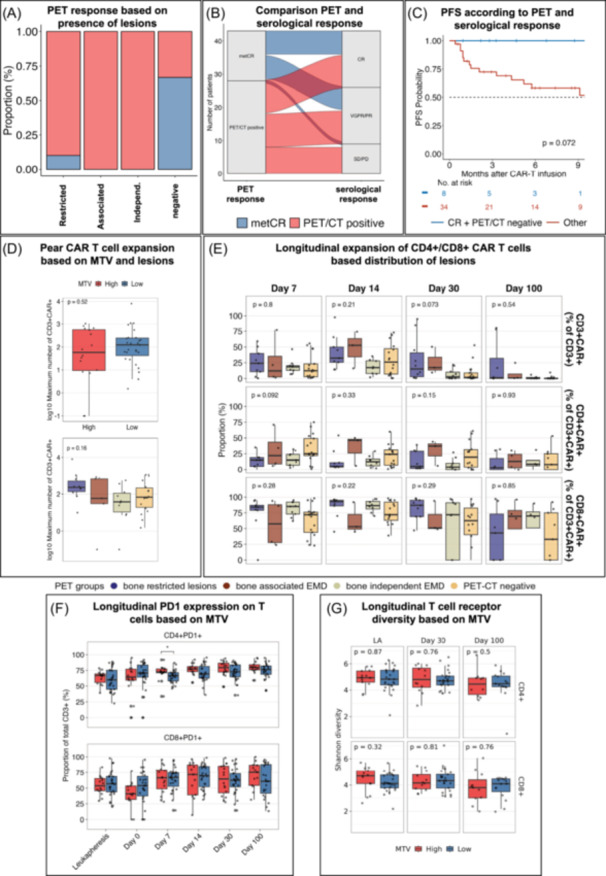
**Impact of PET‐derived remission on outcome and impact on T cell compartment. (A)** Bar plot showing distribution of metabolic CR (metCR in blue) and PET/CT‐positivity (in red) 30 days after CAR T cell infusion based on the presence of bone‐restricted lesions, bone‐associated extramedullary disease (EMD), bone‐independent EMD, or PET‐negativity. **(B)** Alluvial plot showing *n* = 43 patients with available PET/CT after CAR T cell re‐infusion and their response assessment according to IMWG criteria. **(C)** Progression‐free survival (PFS) based on remission according to PET and serological CR. Double‐negative (metabolic CR and serological CR, in blue) had superior outcomes compared to positive patients (red). *p* value was obtained using the log‐rank test. **(D)** Box plots showing peak CAR T cell expansion according to MTV (top) as well as type of lesions (bottom). **(E)** Box plots of CD3+CAR+/totalCD3+, CD4+CAR+/CD3+CAR+, and CD8+CAR+/CD3+CAR+ after CAR T cell infusion between EMD groups. **(F)** Box plots of longitudinal PD1 expression in CD4+ (top) and CD8+ (bottom) T cells in patients with high (red) and low (blue) metabolic tumor volume (MTV). **(G)** Longitudinal assessment of Shannon diversity indices in CD4+ (top) and CD8+ (bottom) T cells between high‐ and low‐MTV patients. Multigroup comparison was performed using the Kruskal–Wallis test. Pairwise comparisons were performed using the Wilcoxon‐rank sum test.

After investigating the impact of baseline PET/CT findings on PFS, we studied whether PET/CT provides complimentary information to serological remission after CAR T cell therapy. Follow‐up PET/CT was available for 43 patients on day 30 post‐infusion; among these, three patients did not have baseline PET/CT imaging performed before treatment. None of the bone associated or bone independent EMD patients, and only one patient with bone restricted FL turned PET/CT negative (Figure 3A). Overall, we observed notable discrepancies between serological disease assessment and PET/CT findings at day 30 post‐infusion. Specifically, among patients classified as being in serologic CR, 52% exhibited persistent metabolic activity detectable in the BM or at FL sites by PET/CT. Conversely, metabolic complete remission (metCR), defined as the absence of detectable metabolic activity, was observed in 37% of patients. Of these metCR patients, 69% concurrently met criteria for serological CR, whereas 25% exhibited very good partial remission or partial remission (VGPR/PR), and 6% displayed stable or progressive disease (SD/PD) according to serological evaluation (Figure 3B). MetCR was associated with a trend toward improved PFS (log‐rank test, *p* = 0.06, Figure S6). Double‐negative patients (metCR and serological CR) showed a trend toward improved PFS (*p* = 0.07, Figure 3C).

### Impact of metabolic tumor volume and EMD on CAR T cell expansion and T cell exhaustion

Next, we investigated whether tumor burden identified by PET/CT has an impact on CAR T cell expansion. There were no significant differences in peak expansion of CD3+CAR+T cells between MTV high and low cases (*p* = 0.52) as well as EMD groups (*p* = 0.16, Figure 3D). Moreover, longitudinal assessment of the proportion of total CD3+CAR+, CD4+CAR+, and CD8+CAR+ cells did not reveal significant differences between EMD groups (Figure 3E). There were also no significant differences in PD1‐expression levels on T cells, except for an increased proportion of CD4+PD1+ on day 7 in MTV high patients (Figure 3F). Interrogation of TCR diversity demonstrated similar Shannon diversity indices at all time points (Figure 3G).

## DISCUSSION

This study demonstrates the pivotal role of PET/CT in assessing prognosis for patients with RRMM treated with commercially available BCMA‐directed CAR T cell therapies. By evaluating PET/CT findings before and after treatment, we elucidated the complex interplay between tumor burden, metabolic activity, and patient outcomes, revealing novel insights into the stratification of high‐risk individuals and potential avenues for optimizing therapeutic interventions.

Patients presenting with bone‐independent EMD at baseline were found to have markedly inferior PFS compared to other PET/CT‐defined lesion groups, corroborating prior studies linking EMD with adverse outcomes in ASCT.^20^ Although neither the number of PET‐positive FLs nor osteolytic lesions on CT influenced outcomes, total MTV proved to be a predictor of early relapse, highlighting the critical value of volumetric tumor burden assessment over lesion enumeration.

Nonetheless, interpreting PET/CT imaging remains subject to inter‐observer variability, and current evidence is limited, with only one real‐world study and one clinical trial of an academic CAR T cell product using PET/CT.^9^, ^18^ Moreover, standardized PET/CT assessments in real‐world clinical practice are presently lacking. The incorporation of the IMPeTUs classification allowed for a standardized interpretation of PET/CT findings. Our analysis revealed that patients with diffuse BM uptake (>Deauville 3) experienced significantly poorer outcomes. Combining IMPeTUs‐defined risk factors—EMD and marrow infiltration—proved highly effective in stratifying patients, enabling the identification of individuals at risk for early relapse or extended survival.

Follow‐up PET/CT imaging revealed a discordance between serologic and metabolic remission in a substantial proportion of patients. Only 37% of patients achieved metCR, emphasizing the value of PET/CT in detecting residual disease that may not be captured by traditional biochemical markers. Double‐negative patients exhibited a trend toward improved PFS, suggesting that combined remission status may serve as a more precise indicator of durable responses. Nevertheless, more studies are needed to evaluate the optimal time point to perform PET/CT after CAR T cell therapies. The persistence of metabolic activity in bone‐independent and bone‐associated EMD after therapy further underscores the aggressive nature of these lesions and the challenges they pose for CAR T cell eradication.

A notable limitation of our study is the timing of PET/CT evaluation at day 30, which may be too early to fully capture disease response or progression in patients treated with CAR T cell therapy. Recent data indicate that extramedullary relapse occurs frequently (>50%) in this patient population after CAR T cell therapy,^21^ emphasizing the necessity for additional, regular PET/CT assessments at later time points, such as day 100 and beyond. Future studies incorporating systematic PET/CT monitoring at defined intervals are essential to establish optimal timing and frequency, potentially improving the detection of extramedullary relapse and enhancing clinical outcomes for this high‐risk group.

Under the hypothesis that a high tumor burden is associated with increased antigen exposure,^22^ we interrogated whether the fitness of the T cell compartment was influenced by tumor burden or EMD. We did not find significant differences in CAR T cell expansion, PD1 expression, or TCR diversity based on PET/CT findings, indicating that T cell exhaustion measured in peripheral blood does not account for the inferior responses observed. Dismal outcomes might rather be explained by local tumor heterogeneity, insufficient trafficking or inactivation of CAR T cells due to T cell suppressive tumor microenvironment, as hypothesized in the use of CAR T cells in solid tumors.^23^

Taken together, we demonstrate that pretherapeutic PET/CT identifies patients at risk for adverse outcome after CAR T cell therapies. Based on our findings and our previous analysis of spatiotemporal heterogeneity in MM,^24^ future studies should incorporate the role of MRD testing especially in cases with EMD and investigate whether PET/CT contributes to identifying MRD‐positive cases in cases with serologic complete remissions. Strategies such as concomitant radiation therapy need to be explored to improve outcomes in this high‐risk population.^25^

## AUTHOR CONTRIBUTIONS

Conception and design: Patrick Born, David Fandrei, Hans‐Jonas Meyer, Lars Kurch, and Maximilian Merz. Acquisition of data (acquired and managed patients, provided facilities, flow cytometry, biobanking, in vitro studies etc.): All authors. Analysis and interpretation of data (e.g., statistical analysis, biostatistics, and computational analysis): David Fandrei, Patrick Born, Hans‐Jonas Meyer, Heike Weidner, Sabine Seiffer, Lars Kurch, and Maximilian Merz. Writing, review, and/or revision of the manuscript: All authors.

## CONFLICT OF INTEREST STATEMENT

M. M.: advisory boards/honoraria/research support: Amgen, BMS, Celgene, Gilead, Janssen, Stemline, Springworks, and Takeda. K. H. M.: BMS: consultancy, honoraria; AbbVie: honoraria, research funding; Pfizer: honoraria; Otsuka: honoraria; Janssen: honoraria; Novartis: consultancy. U. P.: Syros: consultancy, honoraria, research funding; MDS Foundation: membership on an entity's Board of Directors or advisory committees; Silence Therapeutics: consultancy, honoraria, research funding; Celgene: honoraria; Takeda: consultancy, honoraria, research funding; Fibrogen: research funding; Servier: consultancy, honoraria, research funding; Roche: research funding; Merck: research funding; Amgen: consultancy, research funding; Novartis: consultancy, honoraria, research funding; AbbVie: consultancy; Curis: consultancy, research funding; Janssen Biotech: consultancy, research funding; Jazz: consultancy, honoraria, research funding; BeiGene: research funding; Geron: consultancy, research funding; Bristol Myers Squibb: consultancy, honoraria, membership on an entity's Board of Directors or advisory committees; Other: travel support, medical writing support, research funding; BMS: research funding. M. J.: Novartis: honoraria; Amgen: honoraria; Pfizer: honoraria; Blueprint Medicine: honoraria; BMS: honoraria; Jazz: honoraria. The remaining authors declare no competing interests.

## FUNDING

This work is supported by a grant from the Deutsche Forschungsgemeinschaft (SPP µbone) and EU consortium CERTAINTY, and Maximilian Merz received financial support by grants from the International Myeloma Society, European Commission (CERTAINTY), the Jose Carreras Leukaemie Stiftung, DFG (SPP µbone), SpringWorks, Janssen, and Roche.

## TRANSLATIONAL RELEVANCE

PET/CT plays a critical role in evaluating patients with relapsed/refractory multiple myeloma (RRMM) undergoing B‐cell maturation antigen (BCMA)‐directed CAR T cell therapy. This study underscores the utility of PET/CT in identifying high‐risk individuals through high metabolic tumor volume (MTV), diffuse BM uptake, and extramedullary disease (EMD). Patients with bone‐independent EMD demonstrated significantly inferior PFS, while metabolic complete remission (metCR) on post‐treatment PET/CT correlated with better outcomes. Importantly, discordance between serological and metabolic remission highlights the value of PET/CT in detecting residual disease overlooked by conventional markers. Our study advocates for integrating PET/CT into treatment algorithms to stratify risks and refine monitoring strategies, thereby optimizing therapeutic interventions.

## Supporting information

Supporting Information.

## Data Availability

For original data, please contact maximilian.merzr@medizin.uni-leipzig.de.
