# Supplementary material for: Prognostic significance of PET/CT for CAR T cell therapy in relapsed/refractory multiple myeloma
Source: Hemasphere. 2025 Jun 15;9(6):e70159. doi: 10.1002/hem3.70159 (PMC12167625; doi:10.1002/hem3.70159)
Supplement: Supplementary file 1 — Supporting Information. [file HEM3-9-e70159-s001.docx]

Supplemental material to Born et al.,

***Prognostic significance of PET/CT for CAR T cell therapy in relapsed/refractory multiple myeloma.***

**Supplemental Figure 1**

Correlation between number of PET-positive focal lesions (x-axis) and metabolic tumor volume (mTv, y-axis). Each dot is one sample and colors correspond to EMD groups.

**
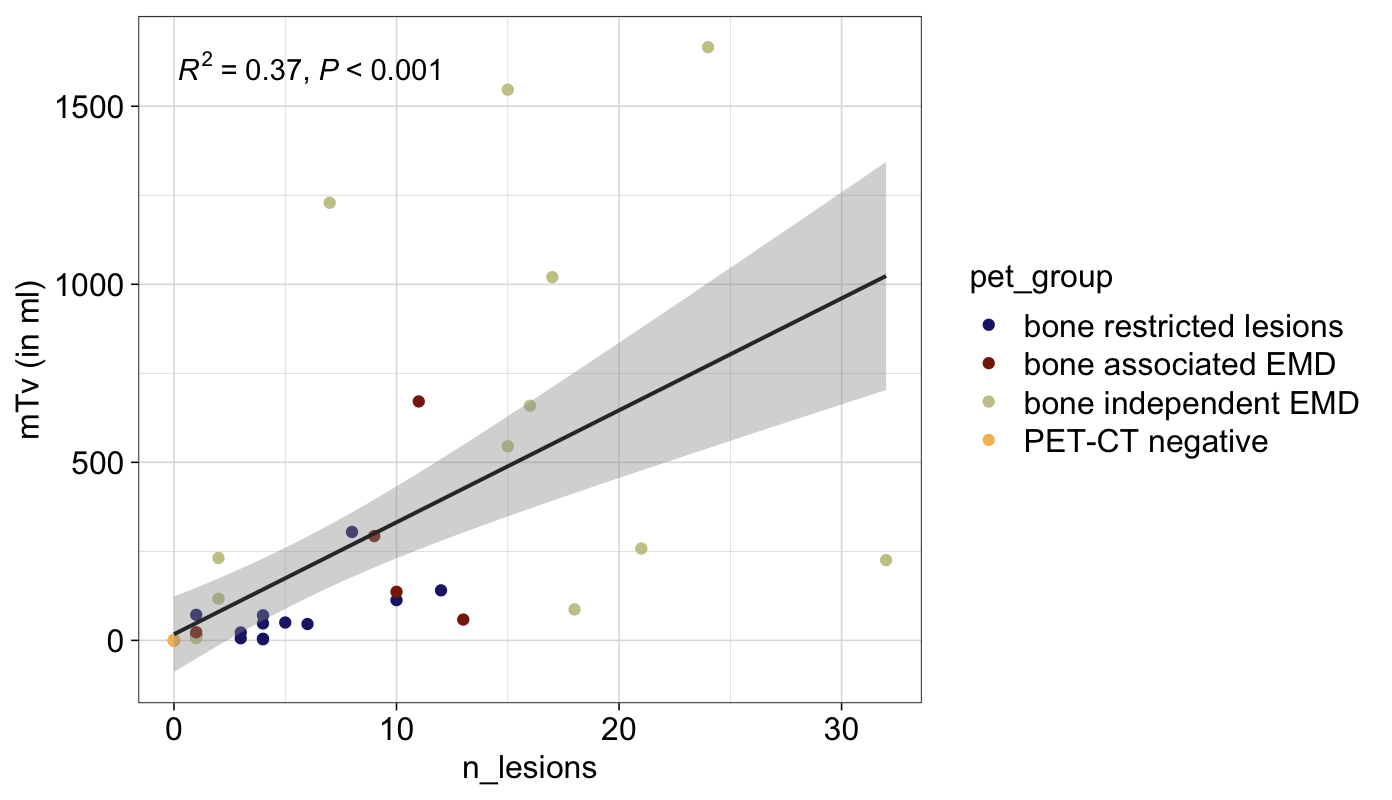
**

**Supplemental Figure 2**

Association between findings from PET/CT and (A) remission 4 weeks after CAR T cell infusion as well as type of progression.


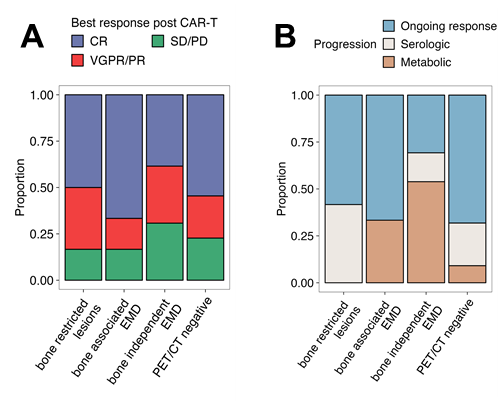


**Supplemental Figure 3**

Association between findings from PET/CT and high-risk cytogenetics (Cytogen., defined as gain 1q21, t(4;14), t(14;16) or del17p), MyCARe and R-ISS scores.


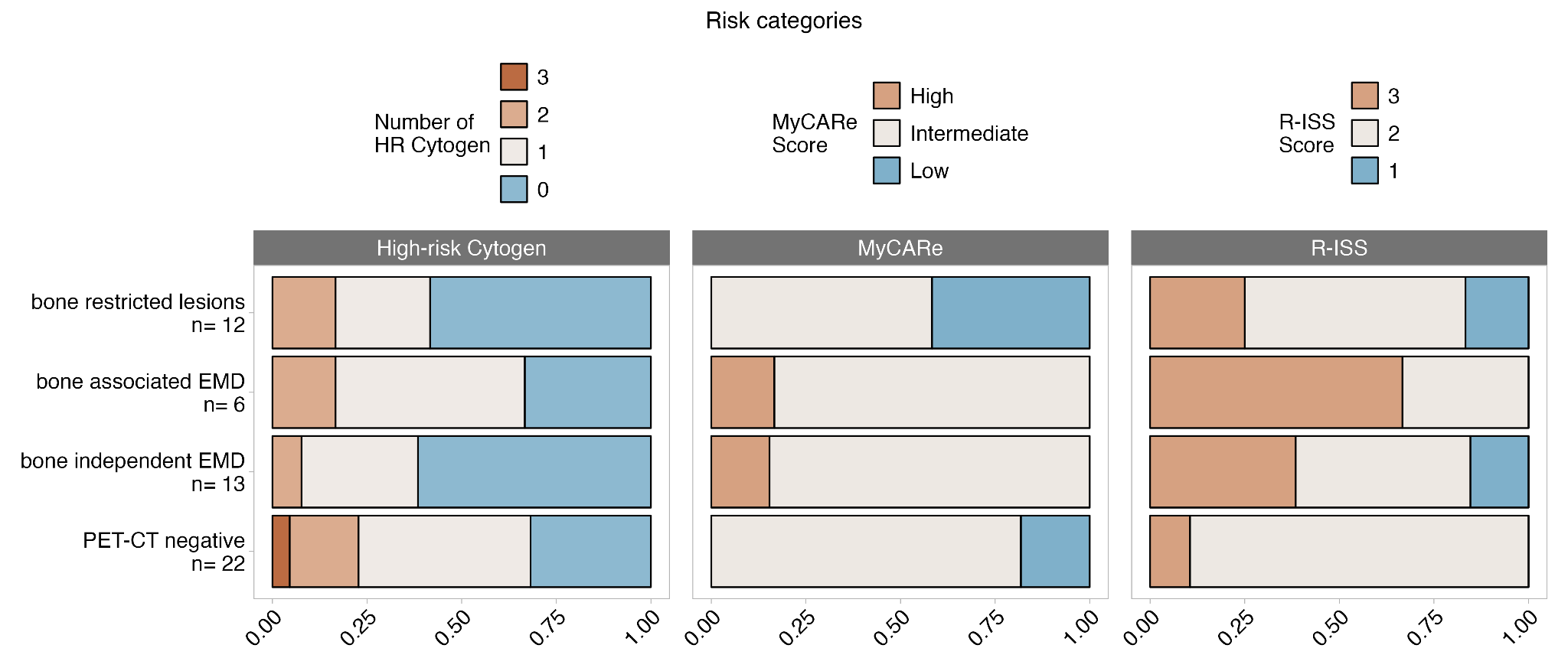


**Supplemental Figure 4A**

Forest plot of univariate COX-PH regression models for PFS.


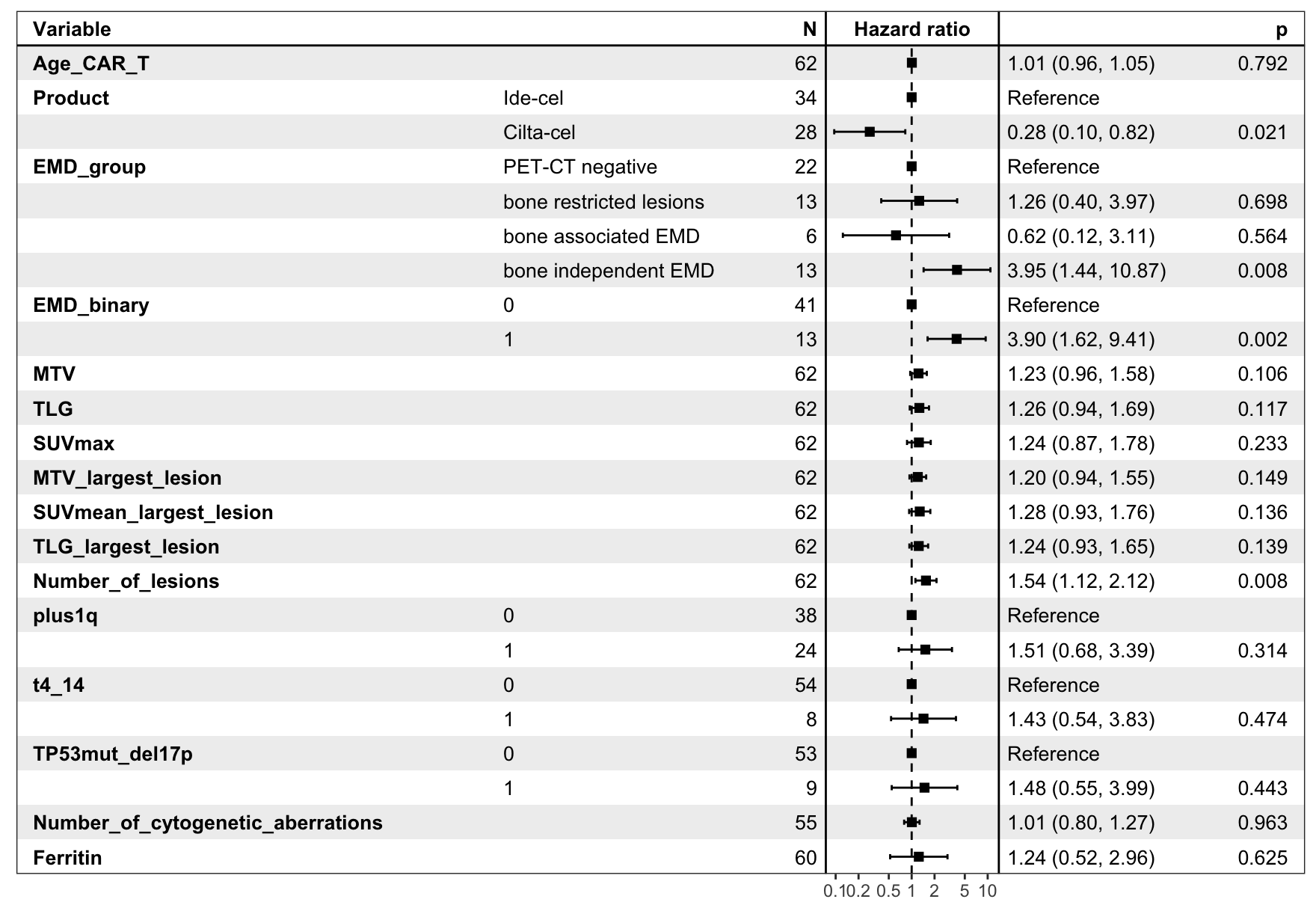


**Supplemental Figure 4B**

Forest plot of a multivariate COX-PH model including type of CAR T product, number of FL, binary EMD status and an interaction term of number of FL * EMD status. Log hazard ratios for PFS are plotted. Hazard ratios, 95% confidence intervals and p-value obtained using the Wald-test are stated.


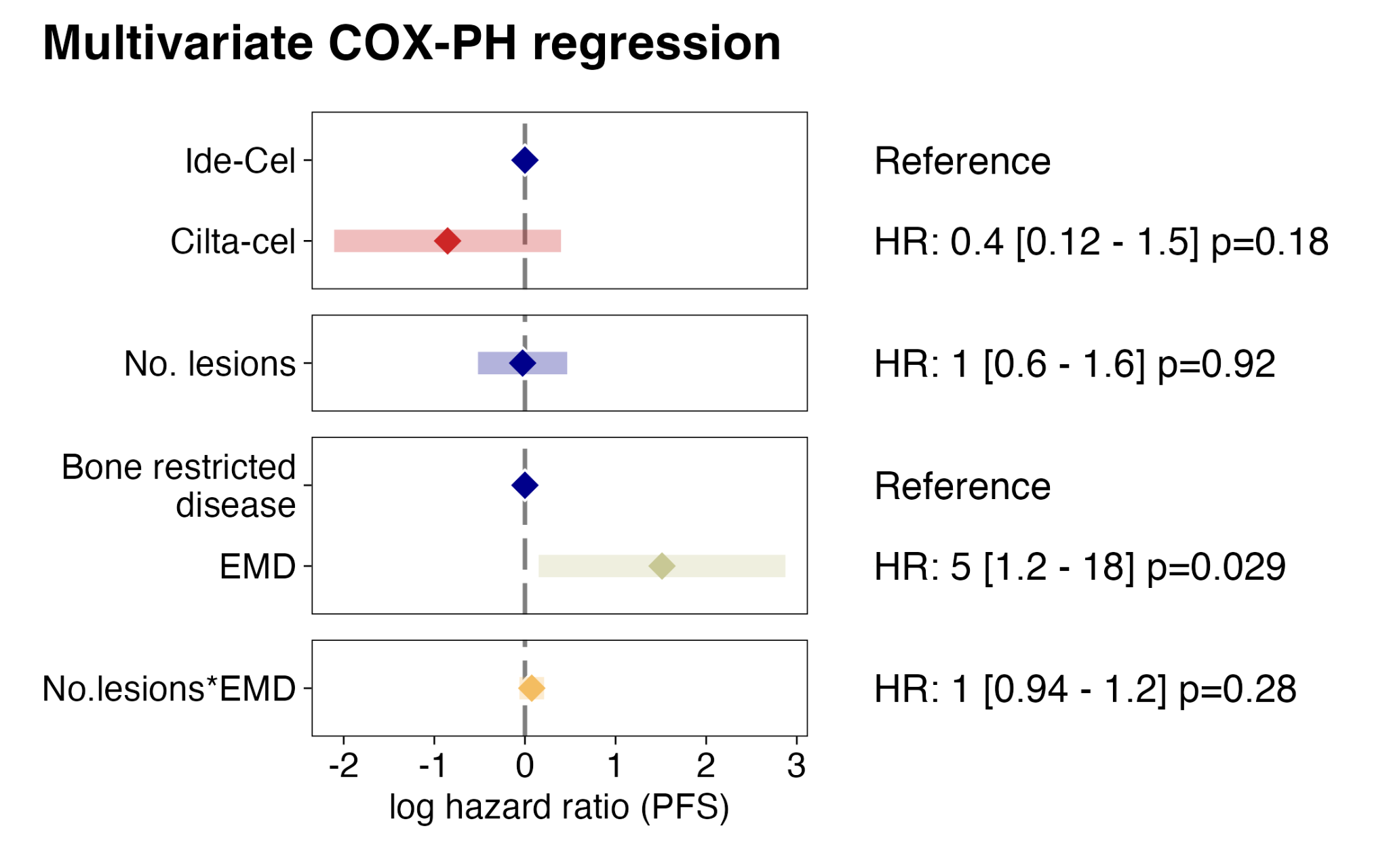


**Supplemental Figure 5**

Association between findings from PET/CT and occurrence as well as severity of cytokine release syndrome (CRS). Toci – Tocilizumab.


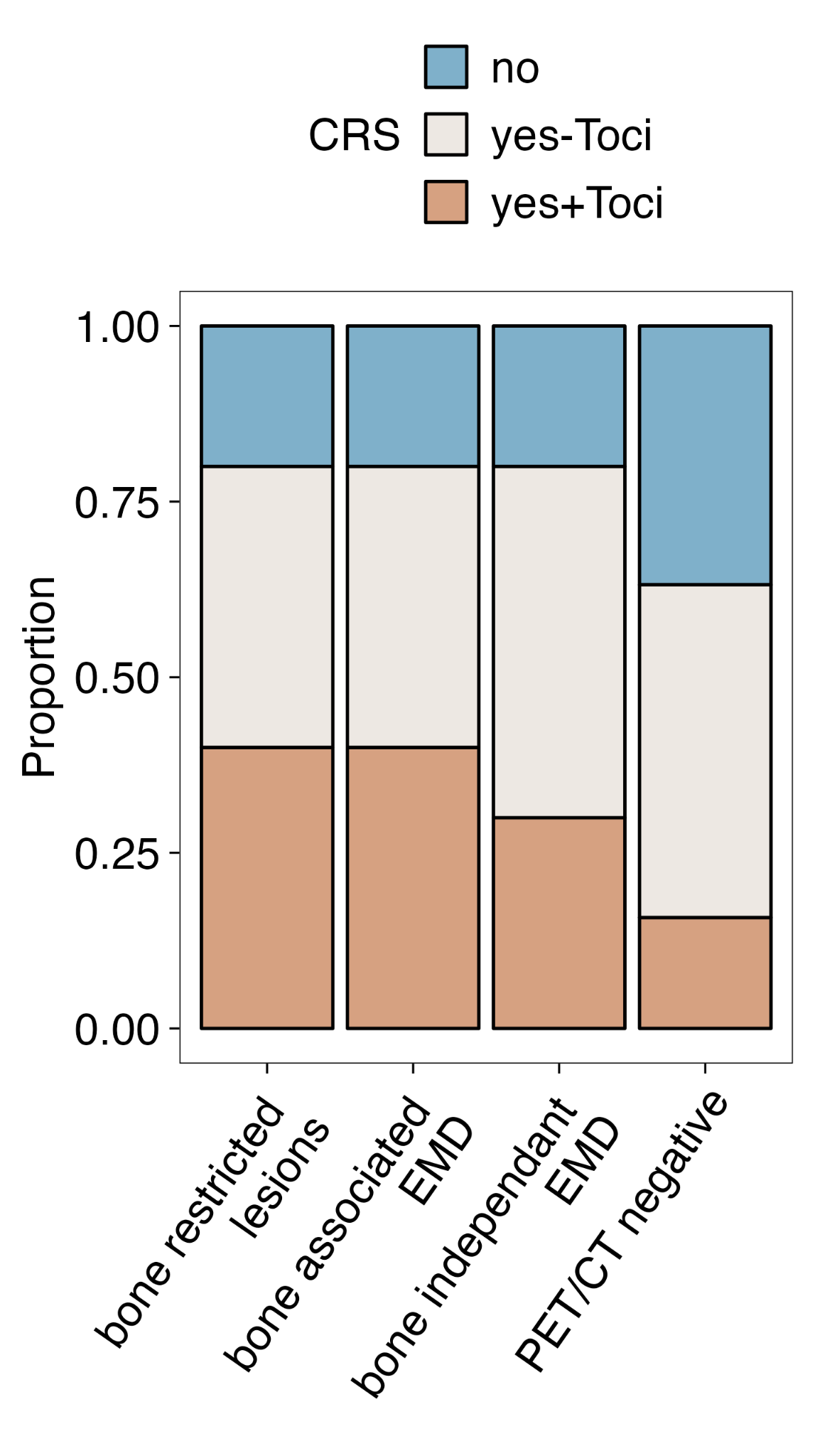


**Supplemental Figure 6**

Association between findings metabolic complete response (metCR) from PET/CT and progression-free survival (PFS).


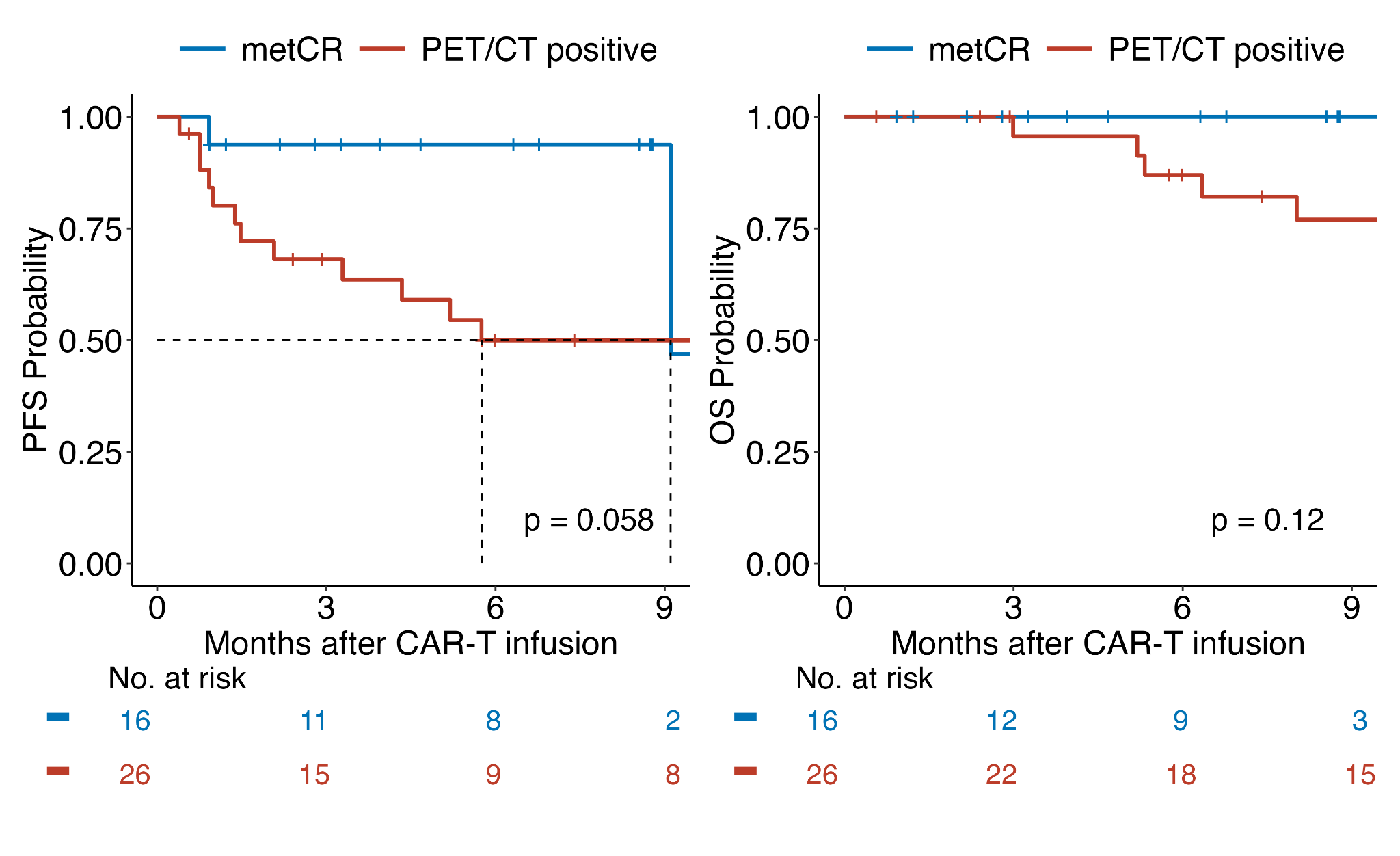


**Supplemental Figure 7**

Delineation of a focal lesion and manual SUV cut-off adjustment to fit the actual lesion volume. The software Hybrid3D viewer calculated mTv based on the relative difference of the SUV in all dimensions surrounding a marked zone of interest.

1. SUV cut-off 5,0 2. SUV cut-off adjusted to account for

actual lesion size


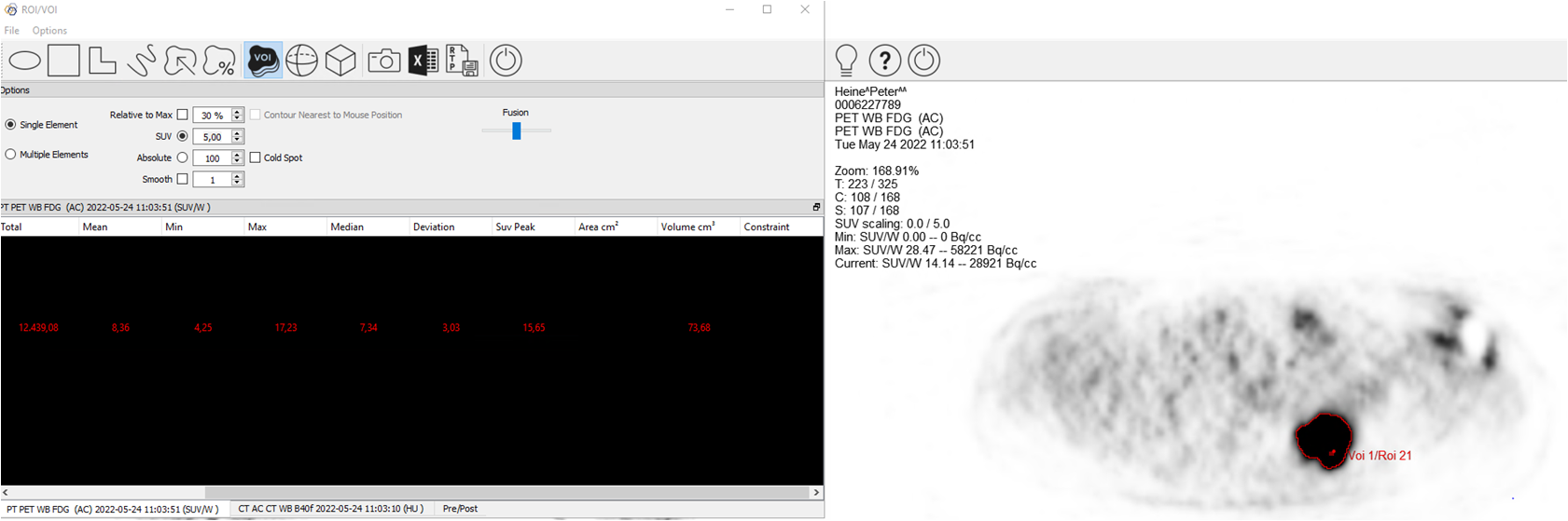

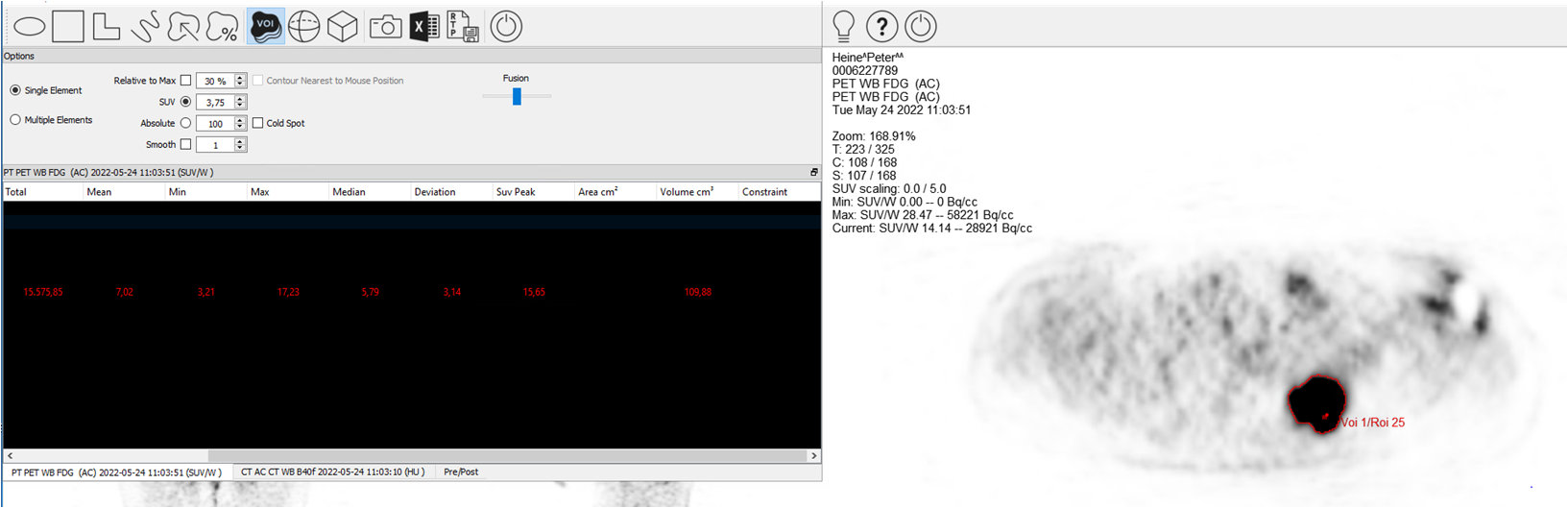


|  | **SUV cut-off** | SUV mean | SUV min | SUV max | **MTV (in cm^3)** |
| --- | --- | --- | --- | --- | --- |
| **1** | **5,0** | 8,36 | 4,25 | 17,23 | **73,68** |
| **2** | **3,75** | 7,02 | 3,21 | 17,23 | **109,88** |
